# Death of Prof. Warner

**Published:** 1847-07

**Authors:** 


### DEATH OF PROFESSOR WARNER.

We regret to have to announce the death of Dr. Augustus S. Warner, Professor of Surgery in Hampden Sidney College, which took place in May last. Prof. Warner had the reputation of being an intrepid and successful surgeon, and able and eloquent lecturer. He died comparatively young.

